# National Dentist Day of India: When and Why?

**DOI:** 10.7759/cureus.104863

**Published:** 2026-03-08

**Authors:** Viswanathan Shanthi, Kaliyamurthy Sivaguru, Nagappan Ragavendran, Thangadurai Maheswaran, Karunamoorthy Vennila

**Affiliations:** 1 Oral Pathology and Microbiology, Tamil Nadu Government Dental College and Hospital, Chennai, IND; 2 Prosthodontist and Implantologist, No. 1 Dental Unit of the Assam Rifles, Shillong, IND; 3 Conservative Dentistry and Endodontics, JKKN Dental College and Hospital, Komarapalayam, IND; 4 Oral and Maxillofacial Pathology, Adhiparasakthi Dental College and Hospital, Melmaruvathur, IND; 5 Periodontology, Vivekanandha Dental College for Women, Namakkal, IND

**Keywords:** father of indian dentistry, indian dental association, indian dental history, national dentist day, rafiuddin ahmed

## Abstract

Dentist Day is celebrated to honor the dental profession and to enhance public awareness of oral health. In the United States, this observance occurs on March 6. India informally adhered to this date and tradition until 2015. However, during the Central Council Meeting of the Indian Dental Association on January 17, 2016, in Jaipur, December 24 was officially designated as the National Dentist Day in India. This date commemorates the birth anniversary of Padma Bhushan Dr. Rafiuddin Ahmed, a pioneer of modern dentistry in India. Despite this official declaration made a decade ago, a substantial portion of the Indian media and numerous dental professionals continue to observe March 6, indicating a notable gap in professional communication.

## Editorial

Introduction

The historical evolution of every profession significantly influences its current identity, and dentistry in India is no exception to this. The transformation of dentistry from ancient oral care practices to a regulated healthcare profession is a narrative rich in detail [1], yet it is often underappreciated. Central to this narrative is Padma Bhushan Dr. Rafiuddin Ahmed, widely recognized as the Father of Modern Dentistry in India [2,3]. It is appropriate that the annual day of recognition for the profession is closely linked to this legacy of excellence. However, a notable discrepancy persists: despite the Indian Dental Association (IDA) officially designating December 24 as the National Dentist Day of India in 2016 [3], both mainstream media and a significant segment of the dental community continue to observe the American date of March 6. This editorial examines the life and contributions of Dr. R. Ahmed, the origins of the National Dentist Day, and the rationale behind India's change in date recognition.

Dr. Rafiuddin Ahmed and modern Indian dentistry

Dr. Rafiuddin Ahmed was born on December 24, 1890, in Bardhanpara, East Bengal (now Bangladesh) [3]. Following his early education, he pursued further studies in the United States and obtained a Doctor of Dental Surgery degree from the University of Iowa College of Dentistry in 1915 [2,3]. Upon his return to India in 1919, he encountered a nation that largely lacked formal dental education and regulation. In 1920, utilizing his personal resources, he founded the first dental college in India, the Calcutta Dental College, which initially enrolled only 11 students and later evolved into the institution now known as the Dr. R. Ahmed Dental College and Hospital [4,5]. This college is acknowledged as the first formal dental college in India [2]. In 1949, the institution was donated to the West Bengal Government, exemplifying his dedication to national service over personal benefit [5].

In addition to his contributions to education, Dr. R. Ahmed established the Indian Dental Journal in 1925 and served as its editor for over two decades, providing the profession with its first academic platform [2,5]. He was instrumental in the formulation of the Bengal Dentist Act of 1939 and the Indian Dentists Act of 1948, the latter being the central legislation that brought dentistry under statutory regulation in independent India [4,5]. In 1925, he founded the Bengal Dental Association, which was later evolved into the Indian Dental Association, an organization that now represents dentists across all states [2,5]. In recognition of his contributions, the Government of India awarded him the Padma Bhushan in 1964, marking him as the first Indian dentist to be honored [5].

National Dentist Day of India

The notion of dedicating a specific day to honor the dental profession originated in the United States, where March 6th is recognized as National Dentist Day. This date was informally adopted into Indian calendars, likely facilitated by globalized professional networks among Indian practitioners. Over the years, it has been observed and celebrated in India without any formal policy foundation, becoming established through repetition rather than the intentional selection of the date.

Acknowledging the necessity for a professional identity grounded in its own historical context, the Indian Dental Association took decisive action. During its Central Council Meeting on January 17, 2016, in Jaipur, the IDA officially designated December 24th as National Dentist Day of India, aligning with the birth anniversary of Dr. Rafiuddin Ahmed [3]. This decision was historically appropriate: Dr. R. Ahmed established the first dental college [1,4], founded the IDA [3], influenced the country's regulatory frameworks [5], and advocated for the profession at a time when it lacked institutional support. No individual embodies the essence of dentistry in India more aptly than Dr. R. Ahmed.

Despite the IDA's formal resolution, the prevailing situation remains largely unchanged. Prominent Indian media outlets and several dental organizations continue to recognize March 6th as India's National Dentist Day in India. This indicates a deficiency in professional communication and the widespread dissemination of IDA policy decisions in the country. The dental profession, which relies on precision and accuracy in clinical practice, must apply the same standards to historical narratives. Dental colleges, IDA state branches, and dental journals must play a role in actively and consistently rectifying this misinformation.

Conclusion

Designating December 24 as India's National Dentist Day is not just a mere change of date; it is a powerful act of professional self-recognition that demands attention and action. This day honors Dr. Rafiuddin Ahmed, a visionary whose personal sacrifice and dedication laid the foundation of Modern Indian dentistry. It is imperative that the dental community embraces this declaration with pride and enthusiasm. We must actively propagate this recognition through journals, college events, and social platforms to ensure that Dr. R. Ahmed's monumental contributions receive the national acclaim. Consequently, the dental community should actively promote this recognition through academic and professional platforms, thereby inspiring future generations of dental professionals.
